# A real-time comparison between direct control, sequential pattern recognition control and simultaneous pattern recognition control using a Fitts’ law style assessment procedure

**DOI:** 10.1186/1743-0003-11-91

**Published:** 2014-05-30

**Authors:** Sophie M Wurth, Levi J Hargrove

**Affiliations:** 1 Institute of Bioengineering, Swiss Federal Institute of Technology (EPFL), Lausanne CH-1015, Switzerland; 2 Center for Bionic Medicine, Rehabilitation Institute of Chicago, Chicago, IL 60611, USA; 3 Department of Physical Medicine and Rehabilitation, Northwestern University, Chicago, IL 60611, USA

**Keywords:** Functional performance assessment, Fitts’ law, Myoelectric control, Upper limb prostheses, Pattern recognition

## Abstract

**Background:**

Pattern recognition (PR) based strategies for the control of myoelectric upper limb prostheses are generally evaluated through offline classification accuracy, which is an admittedly useful metric, but insufficient to discuss functional performance in real time. Existing functional tests are extensive to set up and most fail to provide a challenging, objective framework to assess the strategy performance in real time.

**Methods:**

Nine able-bodied and two amputee subjects gave informed consent and participated in the local Institutional Review Board approved study. We designed a two-dimensional target acquisition task, based on the principles of Fitts’ law for human motor control. Subjects were prompted to steer a cursor from the screen center of into a series of subsequently appearing targets of different difficulties. Three cursor control systems were tested, corresponding to three electromyography-based prosthetic control strategies: 1) amplitude-based direct control (the clinical standard of care), 2) sequential PR control, and 3) simultaneous PR control, allowing for a concurrent activation of two degrees of freedom (DOF). We computed throughput (bits/second), path efficiency (%), reaction time (second), and overshoot (%)) and used general linear models to assess significant differences between the strategies for each metric.

**Results:**

We validated the proposed methodology by achieving very high coefficients of determination for Fitts’ law. Both PR strategies significantly outperformed direct control in two-DOF targets and were more intuitive to operate. In one-DOF targets, the simultaneous approach was the least precise. The direct control was efficient in one-DOF targets but cumbersome to operate in two-DOF targets through a switch-depended sequential cursor control.

**Conclusions:**

We designed a test, capable of comprehensively describing prosthetic control strategies in real time. When implemented on control subjects, the test was able to capture statistically significant differences (p < 0.05) in control strategies when considering throughputs, path efficiencies and reaction times. Of particular note, we found statistically significant (p < 0.01) improvements in throughputs and path efficiencies with simultaneous PR when compared to direct control or sequential PR. Amputees could readily achieve the task; however a limited number of subjects was tested and a statistical analysis was not performed with that population.

## Background

The field of myoelectric upper limb prosthetics has been characterized by great advances in both the development of multi-articulate advanced arm systems [1] and robust and efficient control strategies [2,3] for the operation of those devices.

The current clinical standard of care in myoelectric control strategies is an amplitude-based dual site control. In this approach, commonly referred to as direct control (DC), the mean absolute value (MAV) of the electromyography (EMG) signal amplitude is recorded over an agonist–antagonist pair of residual muscles to control two directions of one prosthetic degree of freedom (DOF) [4,5]. DC is configured to provide a 1:1 mapping of the EMG signal amplitude to the speed of prosthesis movement. Because the number of independent muscle sites in amputee subjects is limited, mode-switching (e.g., through co-contraction of the muscle pair) must be implemented to operate additional DOFs [6]. Thus in DC, operation of more than one DOF requires mode switching and use of control sites that are physiologically unrelated to the intended movement, which makes control cumbersome and unintuitive. Further, as DC relies on isolated muscle contractions, EMG cross-talk and muscle co-activation may decrease the sensitivity and efficiency of this strategy.

An alternative control strategy relies on algorithms that recognize features in the complex EMG signal patterns derived from several electrode locations. Such pattern recognition (PR) control systems decipher user intent by classifying feature sets and assigning them to a given motion class, based on the assumption that each pattern describes a state of muscle activation that is consistent and distinct from other states of muscle activations [7]. As such, PR systems separate complex patterns into a discrete number of classes. PR–based control provides a substantial advantage over DC because independent muscle pairs are not required, which potentially enables control of more DOFs. Many different PR implementations have been investigated with different classifiers, feature sets, signal conditioning, or post-processing techniques; a comprehensive overview can be found in [2,8,9]. PR–based systems have been proposed for the control of the next generation of multifunctional upper limb prostheses [10,11].

Many activities of daily living involve simultaneous movement of several DOFs. Both DC and current PR control systems provide only sequential control outputs, preventing users from performing coordinated tasks in a natural way. Currently, simultaneous activation of several DOFs can be clinically achieved with DC, but only in individuals who have undergone a surgical technique called Targeted Muscle Reinnervation (TMR) [12]. During TMR, the residual nerves of the amputated limb are transferred to available muscles in the residual limb, thereby artificially creating additional independent control sites. After TMR, mode switching is no longer required, EMG cross talk is decreased, and simultaneous control of two DOFs is possible. However, even though TMR is growing in popularity, only a small subset of prosthetic users has had TMR surgery. Moreover, TMR has not yet been performed on transradial subjects, who may benefit from being able to combine wrist and hand motions.

Several research groups have studied the possibility to provide simultaneous control to patients without TMR surgery. Kamavuaka et al. estimated the simultaneous and proportional force in two DOFs from intramuscular EMG using artificial neural networks and achieved correlation coefficients of 88% for the force prediction [13]. Muceli et al. estimated the kinematics of hand and wrist with artificial neural networks from high density surface EMG signals of the contralateral limb during mirrored bilateral motion training on able-bodied subjects and achieved estimation accuracies between 79% and 88% for combined movements [14]. Others focus on using PR algorithms to extract simultaneous, proportional control from surface EMG signals. Cipriani et al. showed a 79% classification accuracy for the simultaneous detection of 7 finger motion classes in 5 amputee subjects [15]. In 2013, Young et al. evaluated the ability of three different linear discriminant analysis (LDA) PR control strategies to provide simultaneous proportional control of a myoelectric prosthesis. They found classification error rates of less than 15% for discrete (i.e., 1 DOF) and combined (i.e., 2 DOF) motions using a hierarchical classifier approach [16]. Baker et al. successfully evaluated a LDA PR algorithm for the decoding of combined finger and thumb motions from implanted myoelectric sensor signals in a monkey’s finger muscles in the forearm [17]. The above-mentioned works show the potential of simultaneous control through high performance in offline evaluations. Further work has indicated the feasibility of using simultaneous controls online and encourages investigation of promising techniques in real-time settings. In 2013, Jiang et al. presented a simultaneous control strategy for two DOFs based on the online extraction of the prosthesis wrist kinematics from a muscle synergy matrix that modelled the factorized surface EMG signals into neural input to individual muscles [18]. In an online positioning task with 2 DOFs, subjects achieved very high completion rates and were able to compete with healthy subjects in terms of efficiency and completion time. The same group also compared the above mentioned control strategy [18] to two other myoelectric control algorithms in real-time [19]. Interestingly, although there were differences in offline performance, the real-time performance was similar, underlining the importance of evaluating control strategy performances in real-time, functional tasks. In 2014, Young et al. demonstrated the feasibility of providing real-time simultaneous control of 2 DOFs with surface EMG PR control, by configuring simultaneous motions as additional classes on TMR subjects [20]. The authors showed that the simultaneous strategy outperformed conventional control strategies in a real-time virtual hand positioning task and that subjects preferred to use a simultaneous class 78% of the time during positioning tasks requiring 2 DOFs instead of positioning the hand in two sequential operations.

Myoelectric control strategies are generally evaluated in controlled laboratory environments. Under such conditions, PR–based systems have achieved classification accuracies higher than 96% with no significant difference between the different classifiers investigated [2,21]. Despite their high potential for the control of multi-DOF upper limb prostheses and an increasing interest in the scientific community, translation of PR systems from fundamental research to clinical application has not yet occurred, although initial clinical trials are ongoing. This delay is due in part to the standard practice of evaluating PR control strategies by determining classification accuracies or errors in an offline evaluation during a disconnected state on previously collected data (and not on data collected in real time during an experiment). While useful, these metrics are not sufficient to comprehensively determine the real time clinical usability of PR systems. Furthermore, classification accuracies do not allow for adequate comparison between different approaches to PR control. Identifying the most promising PR strategy for clinical implementation requires a shift from offline error evaluation to functional performance measures. However, current control algorithms are intended for multifunctional and multi-articulate advanced hand and arm systems, which are not always available to researchers. Existing functional tests are not adequate for objective, pre-clinical performance evaluation of such control systems.

To bypass the need for an actual prosthesis, virtual environments have been developed to evaluate the real time functional performance of myoelectric control strategies. Kuiken et al. developed the Motion Test [3], a relatively simple test that prompts subjects to move a virtual prosthesis through its full range of motion for a given DOF, with the virtual limb moving at a constant speed. Simon et al. developed the more challenging Target Achievement Control (TAC) test [22], which requires positioning of a multifunctional virtual limb from an initial target position back into a neutral position. In the TAC test, the speed of virtual limb movement is proportional to the intensity of the muscle contraction. Test difficulty is modified by changing end-point tolerance, and test complexity level is modified by changing the number of DOFs required to position the limb. While the test can only evaluate one difficulty level at a time, it provides a set of informative performance metrics that allow characterization of control strategies beyond classification accuracies. A limitation of the TAC test is the visual aspect of the task; task completion may be delayed due to difficulty in using visual cues to identify which DOF to operate to achieve the required position. Furthermore the TAC test contains proprietary information and is not generally available.

Lack of pre-clinical functional assessment methods for control strategies hinders the comparison of different approaches across laboratories. A challenging, objective, real time assessment is needed to identify the most promising control strategies for efficient translation of PR into clinical application. The TAC test is a close analogy to a so-called Fitts’ law style test. In 1954, Fitts first quantified human motor performance in a series of discrete pointing movements using a center-out target acquisition test [23]. For rapid, targeted movements, Fitts’ law models the trade-off between speed and accuracy by relating the time taken for a pointing movement to the difficulty of the target (eq. (1)).

(1) MT=a+b*ID

Where MT is the time (seconds) required to move the cursor from the center into one target, ID is the difficulty of the target (eq. (2)) and a and b are the coefficients of the linear regression. Fitts’ law has been extensively used to characterize human-computer interactions, such as those with computer mice or joysticks, and was integrated into the international standard for the evaluation of pointing devices (ISO 9241–9) in 2003 [24]. Williams and Kirsch followed this standard to evaluate a neck EMG–based cursor control system for individuals with high tetraplegia [25]. They evaluated the strategy with a comprehensive set of performance metrics using a two-dimensional target acquisition test. While this study assessed EMG-based control of a cursor on a computer screen, the analogy to myoelectric control strategies for upper limb prostheses is clear, and the study proposed a complete and thorough assessment protocol. Scheme et al. evaluated a PR–based myoelectric control strategy in a pseudo three dimensional Fitts’ law test [26]. They investigated a PR strategy capable of differentiating between three DOFs; however, only one motion class was assessed at a time, thus the proposed assessment did not evaluate the capacity of the PR strategy to classify simultaneous movements. The use of a Fitts’ law style test for the evaluation of myoelectric control strategies has been growing; many different implementations have been proposed, and most of them have evaluated sequential control strategies.

The aim of the present work was to design and implement an objective and challenging test, based on the paradigm of Fitts’ law, to assess functional performance of EMG-based control strategies in real time. A two-dimensional Fitts’ law style target acquisition task test (FTAT test) with a comprehensive set of performance metrics was used to evaluate three control strategies: the clinical standard of care (DC); a conventional, sequential PR (seqPR) strategy; and a simultaneous PR (simPR) strategy. The FTAT test was used with both able-bodied and amputee subjects to identify which strategy had the highest potential for controlling next generation of multifunctional prostheses.

## Methodology

### Subjects

Nine able-bodied subjects and two amputee subjects gave informed consent and participated in this study, which was approved by the Northwestern University Institutional Review Board. Each subject participated in three experimental sessions on three different days to perform the proposed FTAT test with each control strategy. Subjects’ demographic information is shown in Table 1.

**Table 1:** Subject demographics

**Subject**	**Age (years)**	**Amputation**	**Recording site**	**TMR surgery?**
S1 – S9	22 – 30	Able-bodied	Right proximal forearm	n/a
S10	65	Transradial (TR)	Residual forearm	N
S11	35	Transhumeral (TH-TMR)	Residual reinnervated upper arm	Y

### Control strategies, setup, and configuration

The source signal for each control strategy was surface EMG. The signals were amplified using a Texas Instruments TI-ADS1299 analog front end system and sampled at a frequency of 1 kHz. The signal was filtered with a 3^rd^ order Butterworth filter with a 20 Hz cut-off to reduce motion artifact. *Direct control* (*DC*): For able-bodied and the TR subject (S10), pre-gelled adhesive bipolar Ag-AgCl electrodes (BioMedical Instruments, Inc.) were placed on the wrist flexors and wrist extensors. Muscles were identified by palpation of the proximal forearm. The TH amputee subject (S11) had previously undergone TMR surgery and had four independent control sites on the upper arm (Table 1). For this subject we used four bipolar electrodes, and no switching method was required. The MAV of the EMG signal was extracted over a 200 ms sliding window and was directly mapped to the operation of one DOF. We prompted subjects to perform contractions of flexors and extensors at a medium level and adjusted gains to enable them to achieve the full range of motion. Thresholds for signal detection were manually set to reduce muscle crosstalk. Sequential operation of two DOFs was implemented by configuring additional thresholds on both channels such that a short co-contraction of flexors and extensors allowed the user to switch between DOFs. The speed of the control output was proportional to the intensity of the muscle contraction [22]. A single reference electrode, serving as a ground for the acquired EMG signal was placed on the styloid process for able-bodied subjects and on the acromioclavicular joint for amputee subjects.

#### *Conventional sequential pattern recognition control (seqPR)*

For able-bodied subjects, six bipolar electrodes were placed in a ring, equidistantly spaced around the proximal forearm at approximately one third of the arm length from the elbow. For amputee subjects, the electrodes were placed around the residual muscle bulge on the distal residual limb. Precise electrode placement for all subjects was not required as the PR technique extracts feature patterns from all EMG signals rather than isolated amplitudes from independent muscle contractions [27]. A LDA classifier was trained using EMG data acquired during a supervised data collection session: EMG signals were collected during four sets of two repetitions of each motion class, during which subjects were shown pictures of the required movements. For able-bodied and the TR subject, the motion classes were hand open, hand close, wrist flexion, wrist extension, and no motion. The TH subject performed elbow flexion and extension instead of wrist flexion and extension, as this DOF was more intuitive to control and is more important at this level of amputation (Table 2). Subjects were instructed to perform contractions corresponding to the indicated motion class for three seconds, starting from no or a very low level of contraction and gradually increasing contraction intensity to a comfortable, self-selected level, corresponding to the intensity of a firm hand shake. The LDA classifier was trained to recognize the EMG signal patterns in four commonly used time domain features (Mean Absolute Value, Number of Zero Crossings, Slope Sign Changes, and Waveform Length [28]) and six autoregressive coefficients (the first 6 parameters a_i_ from the autoregressive model of the EMG activity [29]) that were extracted in analysis windows of 250 ms with an overlap of 200 ms. Using analysis windows of up to 250 ms has been shown to produce an acceptable trade-off between controller delay and classification accuracy [30]. Proportional control was derived in each window to provide an output speed to the corresponding class [31]. Post-processing of the signal included a decision-based velocity ramp that was shown to reduce the effect of misclassification on the controllability of the system [32]. The LDA classifier was trained using all the data collected during the supervised image-guided sessions.

**Table 2:** Mapping of limb motion to cursor control

**Subject**	**Cursor left**	**Cursor right**	**Cursor up**	**Cursor down**
Able-bodied & TR (S10)	Wrist flexion	Wrist extension	Hand open	Hand closed
TH-TMR (S11)	Hand closed	Hand open	Elbow flexion	Elbow extension

#### *Simultaneous pattern recognition control (simPR)*

Simultaneous pattern recognition control was implemented using a similar approach to a parallel classification strategy proposed by Young et al. [16]. In this approach, one LDA classifier is built for each DOF, allowing for independent classification for each DOF and therefore parallel operation of both DOFs. Six bipolar electrodes were placed as described for seqPR, and signal processing (i.e., filtering, feature extraction, classifier training) was identical to that described for seqPR. In the image-guided data collection sessions, subjects performed discrete and combined motions in four sets of two repetitions each. Proportional control and post-processing was configured in the same way as for seqPR. Each classifier was built with all data from combined motions, discrete motions, and the no-motion class. Motion classes were as for seqPR: hand open/close, with wrist flexion/extension (for able-bodied and the TR subject), or elbow flexion/extension (for the TH subject) (Table 2).

### Fitts’ target acquisition task (FTAT)

**Figure 1:**
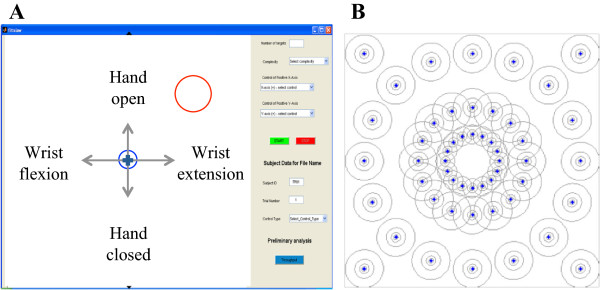
**Fitts’ target acquisition task (FTAT) test. (A)** MATLAB (The MathWorks, Inc.)-based graphical user interface (GUI) for the Fitts’ target acquisition task (FTAT) test. Subjects were prompted to move the blue cursor from the center (blue circle) into the target (red circle) using one of three EMG-based control strategies (See Table 2). **(B)** Widths and locations of all possible targets.

A two-dimensional center-out target acquisition task test was created following the principles of Fitts’ law testing for rapid aimed movements. The goal of the test was to control a cursor in two-dimensional Cartesian space and to steer it from the center of the screen to a circular target that appeared with a given radius at a given location. Figure 1A shows the graphical user interface (GUI), programmed in MATLAB (The MathWorks Inc., Natick, MA), in which subjects controlled the cursor. Figure 1B shows all possible target locations with the two types of targets: on-axis, requiring the operation of one single DOF, and off-axis, requiring the operation of two DOFs. The cursor was controlled using one of the three strategies described above. The mapping of limb motion to cursor movement is shown in Table 2.

A trial began with of the appearance of a target circle on the test space. Subjects were instructed to move the cursor as fast as possible from its initial position in the circle in the center of the space into the target circle and to dwell inside the target for a full second to complete target acquisition. A trial failure was determined by (i) a time-out, (i.e., when target acquisition took longer than 15 seconds); (ii) by excessive overshooting (i.e., when the cursor moved into and immediately out of the target more than 4 times; such targets were considered too difficult), or (iii) when cursor movement was uncontrolled and the cursor bumped into the borders of the test space. Upon trial completion (or failure), the cursor returned to the initial position and the next trial was initiated by the appearance of a new target.

The Fitts’ law test was characterized by a series of target acquisition tasks, each of which was assigned a level of difficulty expressed as the target’s index of difficulty (ID) in bits (eq. (2)).

(2) $$\mathrm{ID}=\log_{2}\left(1+{\;}^{D}{/}_{W}\right)$$

where D is the target’s distance from the center and W is the target’s width as defined by diameter; both D and W are expressed in GUI distance units. The main output metric of the Fitts’ law test is a metric called throughput (TP) in bits/s that describes the information transfer rate of the system (i.e., the control strategy) (eq. (3)):

(3) $$\mathrm{TP}={\;}^{\mathrm{IDe}}{/}_{\mathrm{MT}}$$

where IDe is the ID adjusted for accuracy according to the recommendations of Soukoreff et al. [24], which accounts for the endpoint scatter within each target’s tolerance, as defined by the target width W, of the targeted movements for each condition and subject (eq. (4)).

(4) $$\mathrm{IDe}=\log_{2}\left(1+{\;}^{D}{/}_{\mathrm{We}}\right)$$

where We = 4.133 σ, and σ is the standard deviation of the endpoint positions for a given difficulty condition. As such, the metric throughput incorporates the results from all trials over a wide range of difficulties into one single performance index. It is important to evaluate a strategy over a wide enough range of IDs for an objective evaluation [24]. In [25], Williams and Kirsch used an ID range of 1.58 - 5 bits, while Scheme et al. chose a range of 1.59 - 3.46 bits, showing that higher IDs resulted in higher failure rates [26]. In pilot work, we found that evaluation of control strategies at higher IDs was important for assessing precision control in tasks that required the activation of one or two DOFs [33]. The IDs used with the corresponding target distances (D) and widths (W) are presented in Table 3 and range from 1.273 to 5.047 bits.

**Table 3:** **Range of IDs** (**bits**) **through combination of distances D and widths W** (**GUI distance units**)

**ID (bits)**		**Distance from center (D)**			
		***21.25***	***42.5***	***85***	***120.21***
**Target width (W)**	*3.75*	2.737	3.624	4.565	5.047
	*7.5*	1.939	2.737	3.624	4.09
	*15*	1.273	1.939	2.737	3.172
	*30*	-	1.273	1.939	2.324

### Experimental protocol

After configuration of the control strategy, subjects were first allowed to familiarize themselves with the control strategy by controlling a virtual prosthesis. Subjects were prompted to go through every DOF at low or high speed to verify good control. Further, practice sessions were carried out during each testing session to ensure that subjects had enough time to (1) achieve control of the cursor by mapping muscle contraction to cursor motion on the screen, and (2) reach a plateau in performance. The number of practice trials per session as well as the number of practice sessions was proportional to the complexity of the control strategy and task complexity (i.e. 1 DOF vs. 2 DOFs) (Table 4).

**Table 4:** Practice session protocol

**Practice trials**		**Direct control**	**Conventional PR**	**Simultaneous PR**
**Discrete targets (1 DOF, on axes)**	sessions	1	1	1
	targets/session	11	22	22
**Combined targets (2 DOFs, off-axes)**	sessions	6	6	12
	targets/session	15	30	30

The experimental protocol consisted of six sessions per control strategy. Sessions were separated by rest periods of two or three minutes, depending on muscle fatigue. In every session, 45 targets that required use of either 1 or 2 DOFs were presented to evaluate subjects’ ability to perform both task types with the given control strategy.

### Performance metrics

To comprehensively evaluate each control strategy, we generated a set of five quantitative performance metrics (Table 5).

**Table 5:** Description of performance metrics

**Metric**	**Units**	**Description**
**Throughput**	Bits/second	Index of performance, information transfer rate given through Fitts’ law (eq. (3)).
**Path efficiency**	%	Ratio of Euclidian distance between the origin and the target center to the actual distance the cursor travelled.
**Completion rate**	%	Ratio of successfully completed targets to total number of targets.
**Overshoot**	%	Ratio of overshoots to number of targets. An overshoot was defined by a cursor entry into and exit of the target circle before the dwell time was accomplished.
**Reaction time**	Seconds	Time between target appearance and first move of the cursor.

### Questionnaires

Qualitative evaluation of the control strategies was obtained through a three-part questionnaire (Table 6). Subjects completed a questionnaire after using each control strategy and after evaluating all three strategies. Part I of the questionnaire comprised questions concerning the experimental setup and the test and was administered after the first experimental session. Part II comprised questions about control strategy and was administered after testing each control strategy. Subjects were prompted to answer a last question (part III) after completion of all experiments.

**Table 6:** **Three**-**part questionnaire**: **Subjects were prompted to answer by rating from 1** (**totally disagree**) **to 7** (**totally agree**) **except where otherwise indicated**

	
**Part I**	
1.	I had enough practice to familiarize with the experiment.
2.	I could perceive a difference in difficulty with target distance from center.
3.	I could perceive a difference in difficulty with target width.
**Part II**	
4.	I had enough practice to familiarize with today’s control strategy through VR and GUI practice trials.
5.	The strategy was intuitive to operate.
6.	I managed to stop the cursor movement whenever I wanted.
7.	The mental effort (cognitive burden) was: (*rate from 0* (*nonexistent*) *to 6* (*highest possible*))
8.	The muscle fatigue perceived at the session end was: (*rate from 0* (*nonexistent*) *to 6* (*highest possible*))
**Part III**	
9.	As a strategy to control a myoelectric upper limb prosthesis I would choose: (DC, seqPR or simPR).
	Why?

### Statistical analysis

We assessed the significance of the results for each performance metric with general linear models (GLMs). We built one GLM for each of the five dependent variables (throughput; path efficiency; overshoot; completion rate; and reaction time) and tested for possible interactions between all variables. Session number (one to six); task type (1 or 2 DOFs); and control strategy (DC, seqPR, or simPR) were used as fixed factors and subject was used as a random factor. To further analyze differences between significant effects and interactions, we conducted post-hoc comparisons with a Bonferroni correction factor whenever required. Amputee subject data were analyzed as case studies.

## Results

### Compliance with Fitts’ law

**Figure 2:**
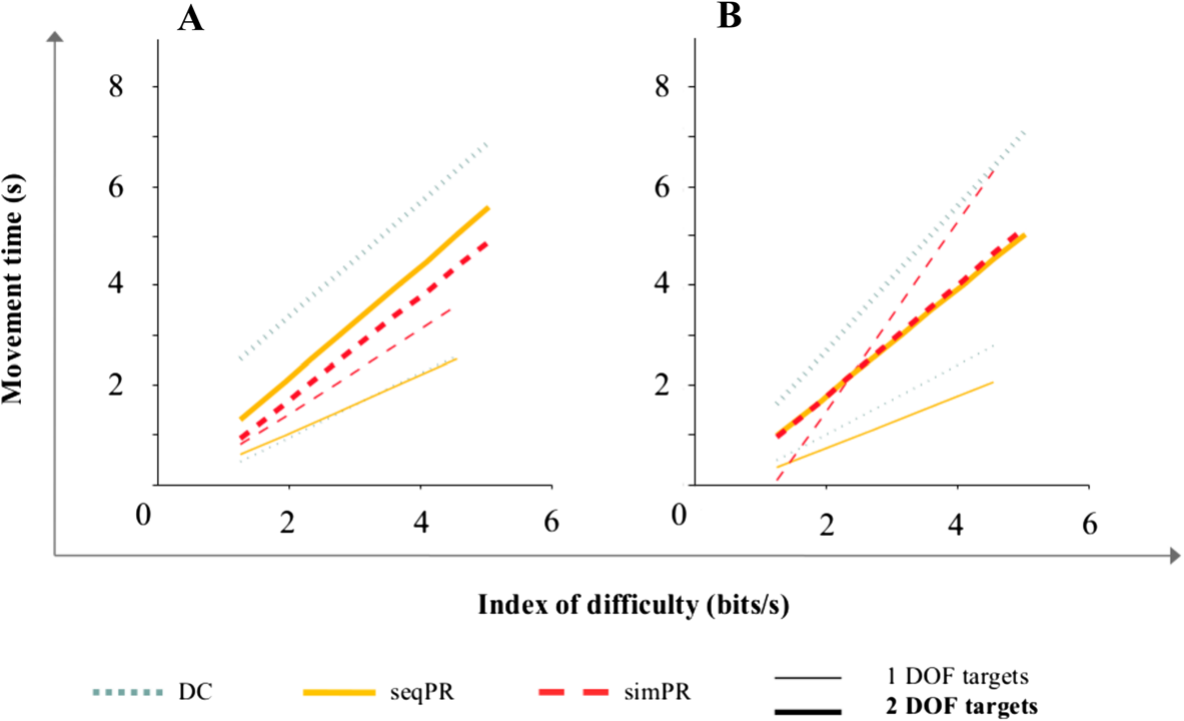
**Fitts’ linear relationship (eq. (**1**)) between movement time and index of difficulty for (A) able-bodied subjects and (B) amputee subjects.** Data is presented for each task type (thin lines represent 1 DOF targets and thick lines represent 2 DOF targets), and each control strategy (DC, seqPR or simPR). **(A)** n = 9 able-bodied subjects. **(B)** n = 2 amputee subjects (S10 and S11).

We obtained a strong linear relationship between movement time MT (s) and index of difficulty ID (bits) across subjects, indicating high conformity of the data to Fitts’ law. The high coefficients of determination R^2^ for both able-bodied and amputee subjects with each control strategy and in each task type (Figure 2) support the validity of the proposed test for EMG-based control strategies. Able-bodied subjects achieved a R^2^ of 0.994 ± 0.004 across control strategies for 1 DOF tasks (Figure 2A; thin lines) and a R^2^ of 0.981 ± 0.003 across control strategies for 2 DOF tasks (Figure 2A; thick lines). Amputee subjects achieved R^2^ = 0.988 ± 0.02 (Figure 2B; thin lines) for 1 DOF targets and R^2^ = 0.9658 ± 0.01 for 2 DOF targets (Figure 2B; thick lines).

### Control strategy performance

#### *Cursor trajectories*

**Figure 3:**
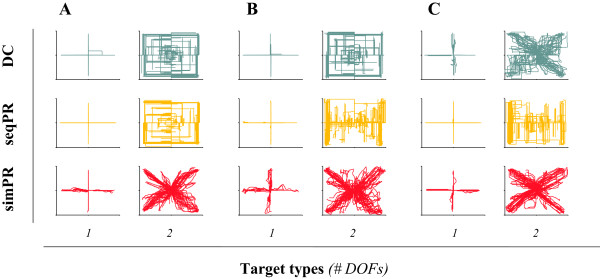
**Cursor trajectories with the three control strategies (DC in green, seqPR in yellow and simPR in red) for (A) one representative able-bodied subject (B) the TR subject (S10), and (C) the TH-TMR subject (S11).** For **(A)**, **(B)**, and **(C)**, the left column represents all cursor trajectories for 1 DOF targets (discrete motions) and the right column represents all cursor trajectories for 2 DOF targets (combined motions).

The goal of the test was to reach the targets as fast as possible using the assigned control strategy. Subjects exhibited a stereotypical cursor profile with each strategy that was consistent across subjects with the exception of the TH subject using DC, where the setup for DC was different due to the additional control sites from prior TMR surgery. Figure 3 shows those profiles for one representative able-bodied subject (3A) and for both amputee subjects (3B and 3C).

#### *Completion rate*

Both able-bodied and amputee subjects had very high completion rates (above 96%) with each control strategy. The GLM for able-bodied subjects yielded target type as a significant factor (p = 0.02); subjects completed significantly less 2 DOF targets (combined motions) than 1 DOF targets (discrete motions). For amputee subjects, there was no difference in completion rates between control strategies or target types.

### Performance metrics

#### *Able-bodied subjects*

**Figure 4:**
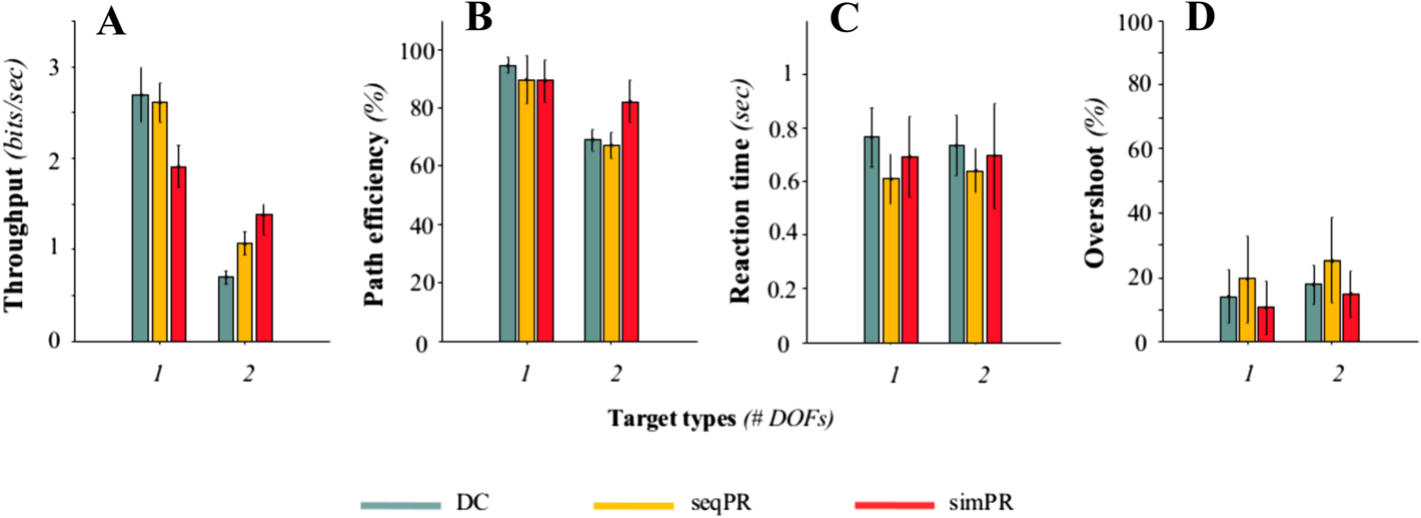
**Performance metrics (Mean ± Std dev.) across able-bodied subjects.** Data is presented for **(A)** throughput (bits/sec), **(B)** path efficiency (%), **(C)** reaction time (sec) and **(D)** overshoot (%) in each of the three control strategies: DC (green), seqPR (yellow) and simPR (red). n = 9 able-bodied subjects.

The performance metrics characterizing the control strategies for able-bodied subjects in the proposed test are summarized in Figure 4. The GLM for each performance metric yielded ‘session number’ as a non-significant main effect. ‘Task type’ was a significant main event for throughput and path efficiency (p < 0.001) and overshoot (p = 0.003). Furthermore ‘control strategy’ was a significant main effect in every metric (p < 0.001 for throughput; p = 0.005 for path efficiency; p = 0.023 for overshoot; and p = 0.013 for reaction time).In 1 DOF targets, able-bodied subjects achieved a similar throughput with DC and seqPR; however, the throughput achieved with simPR was significantly lower (Figure 4A). In 2 DOF targets, throughput was significantly higher using simPR than using seqPR or DC.Path efficiency for 1 DOF targets, in which target acquisition required only a one-directional cursor motion, showed no substantial difference between the three control strategies (Figure 4B). In combined motions, subjects achieved significantly higher path efficiency with simPR, in which paths exhibited a diagonal motion profile (Figure 3A) whereas there was no significant difference between DC and seqPR, both of which were characterized by sequential, boxy motion profiles (Figure 3A).There was no difference in reaction time between task types (Figure 4C). For both task types, seqPR exhibited the lowest reaction time. Subjects exhibited no statistical difference in reaction time between DC and simPR in both task types.For overshoot, the trend across control strategies within each task type was conserved even though subjects completed 2 DOF targets with substantially more overshoots that 1 DOF trials (Figure 4D). Subjects achieved the highest overshoot with seqPR, whereas they overshot significantly less in both task types with DC and simPR.

#### *Amputee subjects*

Data for both amputee subjects is presented separately due to the inherently different characteristics of their respective amputation.

For 1 DOF targets, both amputee subjects had the highest throughput using seqPR (Table 7A), which was substantially higher than the throughput obtained with simPR. While for S11, the throughput obtained with seqPR was also substantially higher than with DC, there was no notable difference between DC and seqPR for S10. For 2 DOF targets, S10 performed with a considerably lower throughput using DC than with either seqPR or simPR; there was no difference between the two PR strategies. S11 exhibited the highest throughput with simPR in 2 DOF targets.

**Table 7:** (**A**) **Throughput** (**bits**/**s**) (**Mean** ± **Std err**.) **and** (**B**) **Path efficiency** (%) (**Mean** ± **Std err**.) **for amputee subjects S10** (**TR**) **and S11** (**TH**-**TMR**)

**(A) Throughput (bits/s)**		**DC**	**seqPR**	**simPR**
**Discrete targets (1 DOF)**	*S10* (*TR*)	2.75 ± 0.17	3.12 ± 0.21	1.36 ± 0.13
	*S11* (*TH* – *TMR*)	2.64 ± 0.24	3.67 ± 0.23	2.11 ± 0.18
**Combined targets (2 DOFs)**	*S10* (*TR*)	0.62 ± 0.01	1.07 ± 0.33	1.08 ± 0.04
	*S11* (*TH*-*TMR*)	1.24 ± 0.04	1.32 ± 0.03	1.63 ± 0.05
**(B) Path efficiency (%)**		**DC**	**seqPR**	**simPR**
**Discrete targets (1 DOF)**	*S10* (*TR*)	93.7 ± 2.12	94.9 ± 1.72	83.7 ± 2.31
	*S11* (*TH* - *TMR*)	90.1 ± 0.23	97.0 ± 0.96	96.3 ± 1.12
**Combined targets (2 DOFs)**	*S10* (*TR*)	63.9 ± 0.81	65.4 ± 0.33	73.7 ± 1.35
	*S11* (*TH*-*TMR*)	71.3 ± 0.8	71.6 ± 0.76	87.7 ± 0.7

**Figure 5:**
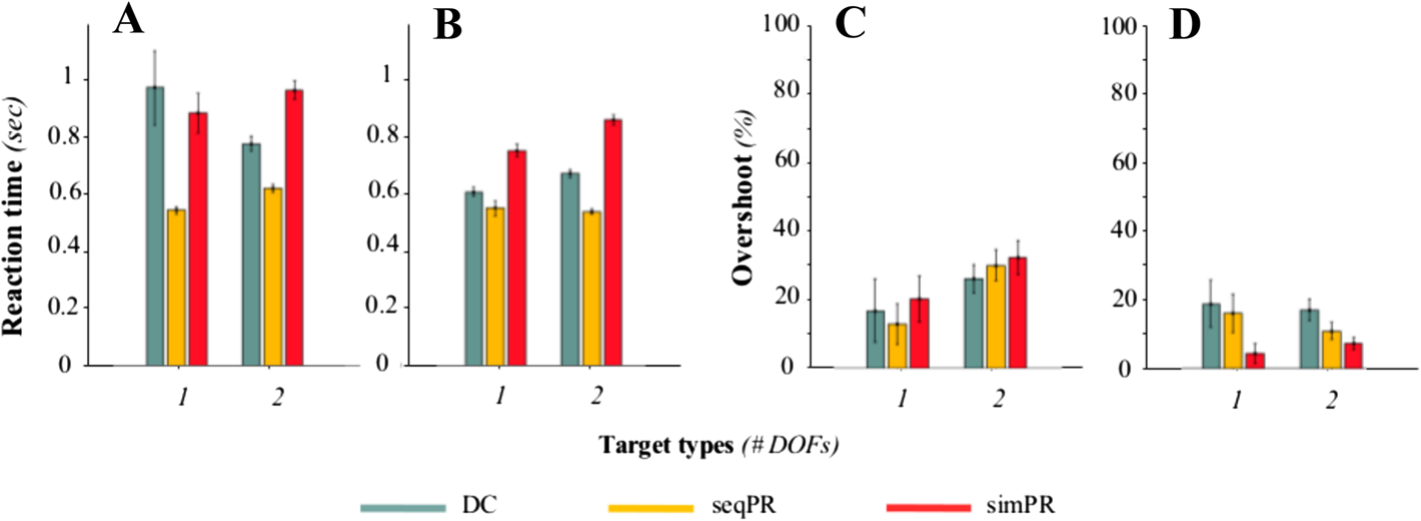
**Reaction time (Mean ± Std err.) and overshoot (Mean ± Std err.) for amputee subjects S10 and S11.** Data is presented for reaction time in **(A)** for the TR subject (S10) and in **(B)** for the TH-TMR subject (S11); for overshoot in **(C)** for the TR subject (S10) and in **(D)** for the TH-TMR subject (S11) in each of the three control strategies: DC (green), seqPR (yellow) and simPR (red).

Path efficiency (Table 7B) was generally high for both amputee subjects in discrete targets. S10 had notably lower path efficiency with simPR than with both other control strategies and S11 had considerably lower path efficiency with DC than with both others. For 2 DOF targets, both amputee subjects achieved the highest path efficiency with simPR while for both, the two sequential control strategies (DC and seqPR) had similar path efficiencies.Both amputee subjects had fastest reaction times with seqPR (Figure 5A for S10, Figure 5B for S11). For both subjects there was no substantial difference in reaction time between DC and simPR.Both amputee subjects had high variability in terms of overshoots (Figure 5). For S10, there was no substantial difference between control strategies within each task type. S10 overshot more in 2 DOF targets than in discrete targets (Figure 5C). There was no difference between task types for S11; however, this subject overshot considerably less with simPR than with both other control strategies (Figure 5D).

#### *Questionnaire*

**Figure 6:**
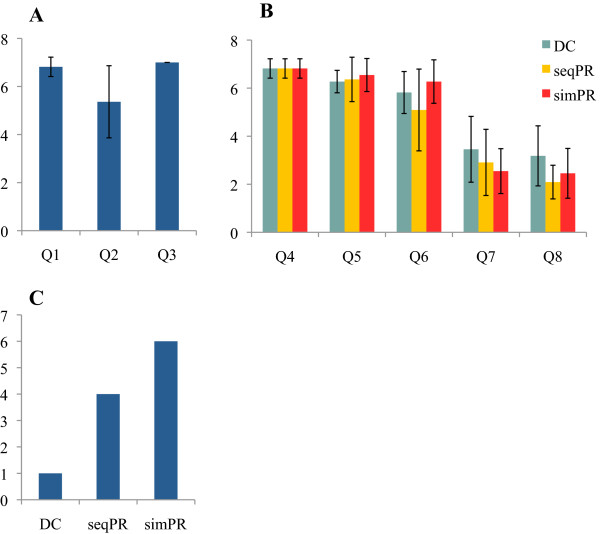
**Results of the three**-**part questionnaire with (A) part I, (B) part II, and part III. (A)** Part I: evaluation (Mean ± Std dev.) of the study design and comprehension in 3 questions with rating (1–7) to (totally disagree - totally agree). **(B)** Part II: evaluation of each control strategy in 5 questions with rating (1–7) to (totally disagree - totally agree) unless otherwise indicated. **(C)** Part III: After completion of the total experiment, subjective evaluation of which strategy subjects preferred. (n = 11: 9 able-bodied subjects, S10, and S11).

Subjects rated the design of the experiment in part I of the questionnaire and strongly agreed that the FTAT test was easy to familiarize with (Q1) and that they could perceive a difference in difficulty with target width (Q3). Subjects did less agree with perceiving a difference in difficulty with target distance from center (Q3) (Figure 6A). Part II concerned the comparison of the three control strategies throughout the FTAT test (Figure 6B). Subjects familiarized equally well with each strategy (Q4) and found that simPR was slightly more intuitive to operate than seqPR or DC (Q5). Subjects reported more difficulties in stopping the cursor on command with seqPR (Q6). Finally, subjects reported highest mental (Q7) and physical (Q8) efforts with DC. Part III of the questionnaire concerned each subject’s preferred strategy for daily use with prosthetics (Figure 6C). Only one subject chose DC, while the others (including both amputee subjects S10 and S11) chose either seqPR or simPR for reasons such as intuitiveness of the control, higher functionality and less physical efforts.

## Discussion

The last two decades have seen extensive efforts in the development of PR control solutions for myoelectric upper limb prostheses. Many strategies have emerged that achieve classification accuracies of more than 96% for sequential control of several DOFs. Despite this success, most of these strategies have not yet achieved clinical application. The delay in implementation is in part due to uncertainty in how to relate offline classification accuracy to real time functionality. Offline performance evaluation is not sufficient to indicate usability, and the lack of appropriate functional tests impedes comparison of different approaches to PR control. Furthermore, few have attempted to compare proposed new strategies to direct control, which is the current clinical standard of care for myoelectric prosthesis control.

We designed and implemented a two-dimensional target acquisition task, based on the principles of Fitts’ law for human motor control, and evaluated it in a clinical study on 9 able-bodied and 2 amputee subjects. To evaluate the capacity of the proposed task to identify promising strategies for the control of the next generation multi-functional prostheses, subjects performed the task using three different EMG-based control strategies. We characterized the interactions between subjects and control strategies through a broad set of performance metrics during two types of task: (i) discrete targets requiring use of one DOF and (ii) combined targets requiring two DOFs. The ability to perform both types of task is extremely important for a natural, robust control of a prosthesis.

We assessed the validity of our methodology by evaluating the conformity of our data to Fitts’ law. Figure 2 shows the extremely high correlation of movement time to index of difficulty across task types and control strategies for both able-bodied (Figure 2A) and amputee subjects (Figure 2B). Our work thus supports the growing use of tests based on the paradigms of Fitts’ law to evaluate EMG-based control strategies in real time [25,26,31]. The different slopes and offsets of the regression lines indicate the differences between the control strategies in terms of both speed and accuracy, which are reflected in the throughput metric (Figure 4A). As a control strategy performance descriptor, the throughput of a system describes information transfer capacity and thus is the average rate of successful message delivery over a communication channel.DC, the current clinical standard of care for myoelectric control, was characterized by high performance in discrete (1 DOF) tasks across able-bodied subjects and both amputee subjects. For these tasks, user intent was efficiently translated into action: cursor movements were both fast and efficient. In combined tasks, subjects had to activate 2 DOFs to acquire the target. For able-bodied subjects and the TR subject, this required a sequential activation of the DOFs that was interrupted by a compulsory co-contraction to switch between modes. This was perceived as both a high mental burden and a cause for increased muscle fatigue, which was efficiently reflected in weaker throughput and lower path efficiencies. S11 (TH-TMR subject) was able to activate each DOF at the same time using DC (because TMR enabled 4 independent control sites); as such S11 was able to move the cursor in diagonal mode, allowing for a higher throughput (i.e., 2 DOF targets were reached faster). Path efficiency for 2 DOF targets was not substantially higher as uncontrolled muscle co-activation hindered precise steering of the cursor into the target (Figure 3C). Despite this difficulty, S11 was able to precisely maneuver the cursor for 1 DOF targets.

For both able-bodied and amputee subjects, seqPR outperformed (or was equal to) DC in nearly every performance metric in both task types. The intuitive nature of seqPR control is reflected in shorter reaction times as well as higher throughput and path efficiency through the possibility to perform seamless sequential control, which is not possible in DC because of the need to switch between modes. Using seqPR, both amputee subjects achieved extremely high throughput in 1 DOF tasks when compared to able-bodied subjects, which is an encouraging result for the clinical implementation of pattern recognition techniques.

Able-bodied and amputee subjects showed very promising results with simPR through the capacity of simultaneously and proportionally activating two DOFs. While this was highly beneficial in 2 DOF tasks, it might have hindered precise activation of one DOF at a time as shown by very low throughputs in 1 DOF tasks. However, although subjects were slower in executing a one-directional motion, they were equally efficient at doing so. A possible solution to the reduced velocity in 1 DOF tasks might be to add gains whenever only one of the parallel classifiers is activated in order to achieve a higher velocity in discrete 1 DOF tasks with low contractions. We noted that subjects needed a lot of practice to learn how to make repeatable and precise contractions. Even though all subjects had reached a plateau in their learning curve before testing, this plateau may be temporary. To obtain the full benefits of simPR, it is possible that a higher mastery level is required, which could be achieved with more practice. The results for simPR suggest great potential for improving control of combined motions, although this strategy requires focused attention during classifier training and an extensive amount of practice. Although performance of discrete motions was not reliable, subjects reported that this strategy was highly intuitive.

The FTAT test was able to identify substantial differences between control strategies and to reveal advantages and shortcomings in performing different task types. Research efforts need to focus on implementing promising strategies and show good control over new generation multiarticulate prostheses. Such devices will enable more than two DOFs to be operable at the same time. While the potential of seqPR and especially simPR (or a different approach to simultaneous pattern recognition control) is clear, the limitations of DC also become apparent: switching through more than two DOFs would be too cumbersome, too unnatural, and impose too high of a cognitive burden.

The FTAT test used is highly modular in terms of difficulty level (e.g. target widths or locations are modifiable), is adaptable to different inputs (e.g., intramuscular EMG, electroencephalography (EEG)), and has as the potential to serve various goals, including comparing different control strategies. Our implementation provides a simple method for direct comparison of PR-based strategies currently being developed with the current clinical standard of care (DC). The setup is very simple and does not contain proprietary information, thus it could easily be implemented to allow direct comparison between different PR control strategies across research laboratories. This work thus contributes to future research efforts on translating pattern recognition into clinical application.

## Conclusion

We were able to use an objective and challenging functional evaluation test to allow comparison of myoelectric control strategies in able-bodied and amputee subjects in a pre-clinical setting. We directly compared in real time, between DC, the current clinical standard of care, and pattern recognition–based control strategies currently being developed to control multi-articulate myoelectric upper limb prostheses. The functional performance evaluation task generates a broad set of performance metrics. The appropriateness of this methodology was validated by finding high conformity of the data with Fitts’ law through high coefficients of determination. The FTAT test allowed us to comprehensively compare functional performance metrics of three control strategies (including throughput (bits/second), path efficiency (%), overshoot (%), and reaction time (seconds)) and revealed significant differences between control strategies. We found that both pattern recognition control strategies outperformed the amplitude-based DC control in coordinated (2 DOF) tasks but that the advantage of a concurrent activation of two DOFs provided by simultaneous PR was offset by a reduced reliability in discrete (1 DOF) motions. Sequential pattern recognition strategy was precise and robust for targets requiring 1 DOF, but depended on a seamless sequential operation to achieve a coordinated task. Both PR strategies were perceived as highly intuitive by subjects whereas DC control was perceived as unnatural and cumbersome to operate, even though efficient for discrete tasks. Our findings will contribute to a convergence of future research efforts to enable translation of PR-based control strategies into the clinic.

## Competing interests

Dr. Hargrove has an ownership interest in a Coapt, LLC, a company working to develop control strategies to control myoelectric limbs. This work was performed prior to the formation of the Company.

## Authors’ contributions

SMW helped in conceiving the study design and experimental protocol, programming the graphical user interface, acquiring, analyzing, and interpreting the data, and drafting the manuscript. LJH helped in conceiving the study design, drafting the manuscript, revising for important intellectual concept, and supervising the study. Both authors read and approved the final manuscript.
